# Self-Reported Work-Related Musculoskeletal Disorders and Associated Factors among Restaurant Workers in Gondar City, Northwest Ethiopia, 2020

**DOI:** 10.1155/2021/6082506

**Published:** 2021-06-07

**Authors:** Habtamu Tegenu, Mulat Gebrehiwot, Jember Azanaw, Temesgen Yihunie Akalu

**Affiliations:** ^1^Gondar City Health Zonal Department, Gondar, Ethiopia; ^2^Department of Environmental and Occupational Health and Safety, Institute of Public Health, College of Medicine and Health Sciences, University of Gondar, Gondar, Ethiopia; ^3^Department of Epidemiology and Biostatistics, Institute of Public Health, College of Medicine and Health Sciences, University of Gondar, Gondar, Ethiopia

## Abstract

**Introduction:**

Globally, work-related musculoskeletal disorders (WMSDs) have resulted in occupational disability and injury. Of these, restaurant workers are among the high-risk professionals usually affected by WMSDs. In Ethiopia, evidence on the burden of musculoskeletal disorder and contributing factors among restaurant workers were very limited. Therefore, this study was aimed at assessing the prevalence of self-reported WMSDs and contributing factors among restaurant workers in Gondar city, northwest Ethiopia, 2020.

**Methods:**

An institutional-based cross-sectional study was conducted from February 2020 to March 2020 among restaurant workers in Gondar city. A two-stage sampling technique was used to choose 633 study subjects. A structured Nordic questioner was used to collect the data. Data was entered into EpiData version 3.1 and exported to Stata version 14.0 for analysis. Both bivariable and multivariable logistic regression analyses were computed. An adjusted odds ratio with a 95% confidence interval was used to measure the association between WMSDs and independent variables. In the multivariable analysis, a *P* value of <0.05 was used to declare a statistically significant association. To check the goodness of fit, the Hosmer and Lemeshow test was used.

**Results:**

The prevalence of WMSDs among restaurant workers in the past 12 months was 81.5% [95% CI (78.18–84.44)]. Attending primary education [AOR: 2.14, 95% CI (1.17–3.90)], attending secondary education [AOR: 1.71 (1.02–2.86)], and job satisfaction [AOR: 1.90, 95%CI (1.13–3.19)] were significantly associated with WMSDs.

**Conclusion:**

In this study, the prevalence of WMSDs among restaurant workers was high. The upper back, lower back, elbow, and wrist were the most affected body parts. Age above 30 years, educational status being primary and secondary, and being dissatisfied by their job were positively associated with a high prevalence of WMSDs.

## 1. Introduction

Work-related musculoskeletal disorders (WMSDs) are one of the common causes of occupational disability and injury among different occupations. It was a major human health problem and loss of work time [1, 2]. Globally, WMSDs are one of the most important public health problems that resulted in a poor quality of life [3, 4], workplace injury, and disability [1, 5]. Moreover, WMSDs cause an approximate loss of 215 billion dollars in the US, 26 billion dollars in Canadian, and 38 billion Euros in Germany [6]. As a result, it has been recognized as one of the leading causes of human suffering, loss of productivity, and economic burdens on society [7].

According to the World Health Organization (WHO), 50–70% of workers developed WMSDs. About 317 million individuals suffer from WMSDs annually and 6,300 individuals died per day [8]. According to the Bureau of Labor Statistics (BLS), 20% of all injuries and illnesses in the workplace and nearly 25% of the annual workers' compensation payment are attributed to back injuries [9]. A more recent report by the national safety council indicated that overexertion is the most common cause of occupational injury, which accounts for 31% of all injuries [9]. In Ethiopia, there is a poor working environment and no strong functioning health and safety system.

In Ethiopia, studies regarding WMSDs are conducted on different industrial workers. However, studies among restaurant workers are very limited. Besides, the attention given by the ministry of labor and social affairs was minimal. Thus, the magnitude of WMSDs among restaurant workers was unknown in the country. Therefore, this study was aimed at determining the magnitude of WMSDs and the associated factors among restaurant workers in Gondar town.

## 2. Methods and Materials

### 2.1. Study Design and Setting

An institution-based cross-sectional study was conducted from February to March 2020 in Gondar city, Amhara Regional State, Northwest Ethiopia, which is located about 750 kilometers (km) from Addis Ababa, the capital city of Ethiopia. The city comprises six subcities (Fasil, Jantekel, Arada, Zobel, Maraki, and Azezo). In Gondar city, there are 101 restaurants and 1309 restaurant workers that provide services to customers. They are working in food preparation, cooking, distribution, food hygiene, service cleaner, and cashier.

### 2.2. Population and Sample

The source population included all restaurant staff in Gondar city and the sample population consisted of randomly selected restaurant employees. Restaurant workers who had a previous history of a vehicle or other work-related accidents, who were physically deformed, and who were pregnant women were excluded.

### 2.3. Sample Size Determination

**Figure 1:**
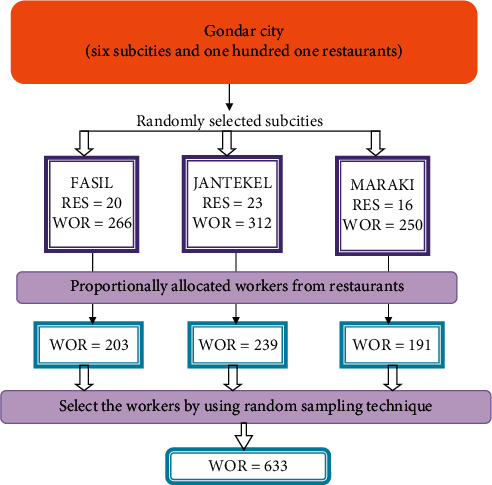
Schematic presentation of the sampling procedure.

The sample size was determined using a single population proportion formula. A 50% proportion, 95% confidence interval (CI), a 5% margin of error, a design effect of 1.5, and a 10% nonresponse rate were used to determine 633 study subjects. A two-stage sampling technique was used to choose the study subjects. All restaurant workers in the selected restaurants who fulfill the inclusion and present during the data collection time were interviewed (Figure 1).

### 2.4. Data Collection Tools and Procedure

A standardized Nordic questionnaire [10] using a face-to-face interview was used to gather musculoskeletal symptoms within the past 12 months. The questionnaire was divided into four parts. These are sociodemographic characteristics, sex, age, religion, educational status, marital status, monthly salary, and work experience; workplace factors, working hours per day, health and safety training, number of customers per day, and work posture; psychosocial factors, job satisfaction and job stress; and individual factors, physical exercise, alcohol drinking, smoking cigarette, body mass index (BMI), job training, and coping mechanisms.

### 2.5. Variable of the Study

In this study, the dependent variable, self-reported WMSDs, was defined as a self-reported pain, ache, or discomfort for all or at least one symptoms in the past 12 months in any part of the neck, shoulder, upper back, lower back, hips/thigh, knee/leg, ankle/foot, elbow, and wrist/hand [11]. Independent variables include sociodemographic factors, age, sex, marital status, educational level, work experience, and monthly income; individual factors, BMI, training, physical activity, smoking, and drinking; workplace factors, job category, adjustable chair and table, workload, work posture, repetitive movement, working hours, safety training, and the number of customers per day; and psychosocial factors, job satisfaction, job stress, and relationship with others.

Good job satisfaction was defined when a restaurant worker responds 32 and above, whereas respondents who respond <32 were classified as unsatisfied [12]. Job stress: a restaurant worker who responds 16 and above was categorized as stressed, whereas respondents who respond less than 16 were classified as nonstressed [13]. Awkward postures (AP) include working with the neck bent more than 30 degrees without support, working with a bent wrist, working with the back bent without support, squatting, and kneeling for two or more hours [14]. Highly repetitive work (RW) is defined as work involving repeating the same motion with less than 30 seconds or no variation every few seconds for two or more hours [14]. Static posture (SP) is defined as a restaurant worker who is sitting or standing in a restricted space for two or more hours without changing positions [14]. Body mass index means weight in kilogram divided by the square of the height in meters (kg/m^2^) [15]. Underweight was defined as BMI <18.50, normal weight was defined as BMI b/*n* 18.50–24.99, and overweight was defined as BMI ≥25.00. Cigarette smoker is defined as smoking at least one stick of cigarette per day [15]. Alcohol drinking is the consumption of any kind of alcohol by restaurant workers at least two times per week [15]. Doing physical exercise is defined as doing any kind of sports activities at least two times per week with a duration of at least 30 minutes [16]. Job training is a set of practices that happen in a specific organizational setting which include adapting and obtaining integrated clusters of values, skills, knowledge, and feelings that lead to essential changes in behaviors of a worker or teams [17]. Ergonomic training is educational actions for credentials by employees, risk factors accountable for musculoskeletal disorders related to work, use of suitable work practices, proper equipment choice, correct use of tools, and adjustments of the workplace [18].

### 2.6. Data Quality Control

An interviewer-administered structured questioner was used. The questionnaire was initially prepared in English and translated into Amharic (the local language). Then, the Amharic version was translated back to English to check for inconsistencies. A pretest was conducted on 32 participants in Debark town restaurants and necessary modifications were made. The reliability of the questionnaire was evaluated and validated. Two-day training was given to the data collectors and supervisors. Moreover, the questionnaires were checked for completeness by the supervisors and the principal investigator daily.

### 2.7. Data Management and Analysis

Data were edited, coded, and entered into EpiData version 3.1 and exported to Stata version 14.0 for analysis. Descriptive findings were presented using frequency tables, graphs, percentages, and proportions. An adjusted odds ratio (AOR) with a 95% confidence interval was used to measure the association between WMSDs and the independent variables. Bivariate logistic regression analysis was used to show the presence of a statistically significant association. The goodness of fit was checked using the Hosmer and Lemeshow test.

## 3. Results

### 3.1. Sociodemographic Characteristics of Restaurant Workers

The response rate of this study was 94%. Of the total respondents, 419 (70.42%) respondents were females. The mean age of the respondents was 23 ± 3 standard deviation (SD). The majority, 524 (88.07%), of the respondents were under 30 years. Of all, 428 (71.93%) were single and 585 (98.32%) were orthodox by religion. The median monthly income of participants was 1,388 ETB with an interquartile range (IQR) of 500 to 8000 birr. Nearly half of the respondents (52.10%) had less than 1,100 ETB salary (Table 1).

### 3.2. Personal Characteristics of Restaurant Workers

Almost all (99.16%) and 494 (83.03%) of the restaurant workers were nonsmokers and nonalcohol drinkers, respectively. Only 105 (17.65%) of restaurant workers perform a physical exercise at least two days per week. About 15% of restaurant workers were obese (Table 2).

### 3.3. Workplace Characteristics of Restaurant Workers

Nearly half (52.4%) of restaurant workers spent more than 8 hours per day at the workplace. Only a few (16.64%) of the participants took more than two breaks to rest per day excluding lunch break. The majority (71.26%) of the respondents spent 7 days per week and 171 (28.74%) of the respondents spent 4–6 days per week. More than half (65.88%) of the participants were working in a static posture for 2 hours (Table 3).

### 3.4. Working Environment and Psychosocial Characteristics of Restaurant Workers

**Figure 2:**
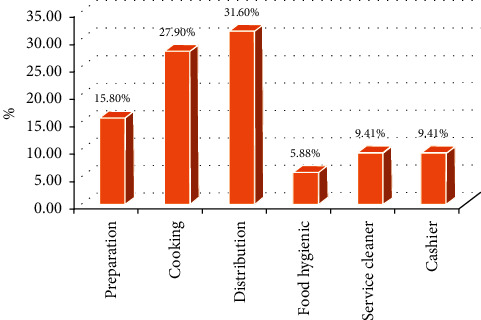
Job category of restaurant workers in Gondar city, Ethiopia, April 2020.

This study showed that 166 (27.9%) respondents were performing cooking tasks. Nearly one-third (31.6%) of the respondents were waiters (Figure 2).

Regarding psychosocial factors, 394 (66.2%) of restaurant workers reported that they had a good work relationship with their colleagues and 432 (72.61%) restaurant workers had good work relationships with their customers. Regarding job satisfaction, 490 (82.35%) of the restaurant workers were not satisfied with their current job. On the other hand, 538 (90.42%) of restaurant workers had job stress (Table 4).

### 3.5. Prevalence of Self-Reported WMSDs among Restaurant Workers

**Figure 3:**
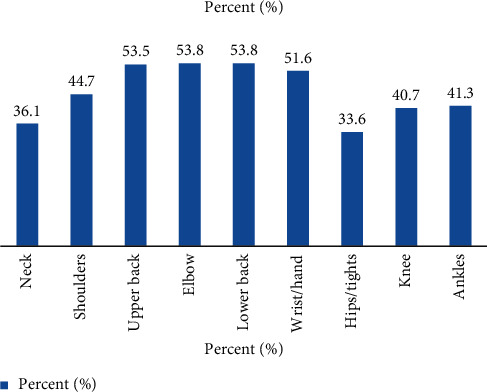
The prevalence of each body part of self-reported WMSDs among restaurant workers in the last 12 months, April 2020.

Out of 595 restaurant workers, 485 (81.5%) had reported pain or ache in any part of the neck, shoulder, upper back, lower back, wrist, elbow, hip/thigh, knee, and ankle for the past 12 months. The most affected body parts include elbow 320 (53.8%), lower back 320 (53.8), upper back 318 (53.5%), and wrist 307 (51.6%) (Figure 3).

### 3.6. Multiple Body Parts (Right and Left Side) WMSDs among Restaurant Workers

Almost all restaurant workers reported multiple body parts (right and left) pain in the shoulder, elbow, hand/wrist, hips/tights, knee, and feet/ankle. Of the total participants who had pain in the shoulder, 266 (44.7%), 147 (24.7%), 84 (14.1%), and 35 (5.88%) had in both shoulders, right and left shoulder, respectively (Table 5).

### 3.7. Self-Adopted Management Strategies for Musculoskeletal Complaints

Restaurant employees had used a variety of coping mechanisms. About 30% of restaurant workers preferred taking sufficient rest as a coping mechanism (Table 6).

### 3.8. Factors Associated with Self-Reported WMSDs among Restaurant Workers

In the bivariate analysis, age, educational status, monthly salary, work experience, BMI, awkward bending and twisting, work repetition, adjustable chair and table, and job satisfaction had a statistically significant association with self-reported WMSDs. In a multivariable logistic regression analysis, age, educational status, and job satisfaction remained statistically significant.

The odds of developing WMSDs among respondents with the age of >30 years were 4.7 [AOR: 4.7, 95% CI (1.62–13.84)] times when compared to respondents under the age of ≤30 years. Moreover, the odds of developing WMSDs among respondents who complete the primary and secondary levels of education were 2.14 [AOR: 2.14, 95% CI (1.17–3.90)] and 1.71 [AOR: 1.71 (1.02–2.86)] times that of their counterparts, respectively. Similarly, the odds of the self-reported work-related musculoskeletal disorder among restaurant workers that were not satisfied were 1.90 [AOR: 1.90, 95%CI (1.13–3.19)] times that of satisfied restaurant workers (Table 7).

## 4. Discussion

In this study, the prevalence of work-related musculoskeletal disorders among restaurant workers in Gondar town within the past 12 months was 81.5% with 95% CI (78.2–84.4). Lower back (53.8%), elbow (53.8%), upper back (53.5%), and hand/wrist (51.6%) pain had the highest prevalence, respectively. Age, educational status, and job satisfaction had significantly associated with WMSDs among restaurant workers in Gondar town.

This finding is in line with a study conducted in Taiwan (84%) among restaurant workers [19]. In contrast, the finding was higher than the study conducted in Iran (70%) [20], Spain (69.2%) [21], Turkey (59%) [22], Bangladesh (78%) [23], and Hong Kong (60%) [24, 25]. The first possible explanation could be the manual-based working process in the current study which may elicit higher exhaustion. The second reason could be the presence of poor health and safety service in Ethiopia compared to the former studies. This could cause inadequate health and safety professionals, inadequate personal protective equipment (PPE), and poor knowledge among restaurant workers and employers. As a result, restaurant workers would be exposed to a low preventive practice and repetitive occurrence of occupational disorders.

The third possible explanation could be a difference in assessment tools. A self-reported administered questioner was used in the current study. However, an ergonomic measurement tool was used in the former study [26, 27]. As a result, the findings from the self-reported questioner could be overestimated or underestimated compared with ergonomic measurements.

Findings are not compared to the local context due to a lack of literature on the area. Besides, the prevalence of specific body parts was explained as follows: in this study, the prevalence of lower back pain was 53.8% (49.76%–57.79%). This study is in line with the study conducted in Taiwan (52.2%) [19]. In contrast, the current study is lower than the study done in Gondar among hotel housekeepers (58.1%) [28]. A possible explanation could be the age difference. In the former study, nearly 54% of respondents were above the age of 25 years [19] and in the current study, only 47% of participants were above the age of 25 years. This is because depletion of bone density begins at the age of 30 years, the risk of lower back pain increased as ages increase. As a result, bone can be easily damaged and developing WMSDs [29].

In this study, the prevalence of upper back pain was 53.5% (49.42–57.46) which was higher than the study conducted in Bangladesh (38%), Nepal (7.5%), and Taiwan (32.7%) [19, 23, 30], respectively. The possible reason might be due to the difference in the study participants, sociodemographic characteristics, and differences in workload. The prevalence of elbow and wrist pain was 53.8% (49.76%–57.79%) and 51.6% (47.56–55.62), respectively. This study was higher than the study conducted among restaurant workers in Taiwan (27.3%) and Nepal (5%) and hotel housekeeping in Gondar town (47.2%) and barbers in Gondar town (29.3%) [19, 30–32], respectively. The possible explanation could be the restaurant workers' tasks require repetitive and forceful movement of the hand as compared with housekeeping and barbers. The difference in socioeconomic and working processes in the study area and Taiwan is quite different. This could cause a high prevalence of WMSDs in the current study.

The other most body parts where the respondents reported pain or discomfort in this study were neck 36.1%, shoulder 44.7%, hips/tights 33.6%, knee 40.7%, and ankle or foot 41.3%. This was higher than the study done in Taiwan, Bangladesh, Spain, and Turkey among restaurant workers and hairdressers in Gondar town [19, 21–23, 33], respectively. The possible explanation could be that restaurant workers' tasks require repetitive and forceful movement of the hand, elevated shoulder, and bending at the back; these working postures induce pain of lower and upper body parts. Besides, the restaurant worker performs tasks in a standing position and this can bring a static load on leg muscle causing pain in this area.

Age above 30 was associated with higher odds of developing WMSDs. This study was supported by studies done in Bangladesh, South India, and South Korea [23, 34, 35], respectively. The possible explanation might be due to the biological structures of the human body, particularly related to muscles, joints, nerves, ligaments, and tendons, which would tend to degenerate as the ages of the workers increase and will induce pain. Another possible reason could be that age causes bone depletion that results in WMSDs [29].

Restaurant workers attending primary and secondary education were at higher odds of developing WMSDs compared with those with above higher education. This finding is supported by a study in Egypt [36]. The study done in Egypt showed that the implementation of the educational intervention significantly improved workers' knowledge and practice regarding work-related musculoskeletal disorders. As a result, the burden of MSDs was low after the educational intervention than before the intervention. Hence, providing training and enhancing their educational status could minimize the burden of MSDs among restaurant workers.

The odds of developing WMSDs among nonsatisfied restaurant workers were higher than satisfied counterparts. This finding was consistent with the study conducted in a Chinese systematic review among the catering industry staff, Greece among cosmetologists, Japan among cookers [37–39], and Gondar town among hotel housekeeping respondents [28]. The possible reason might be that those workers who were dissatisfied with the working condition and environment might develop work-related stress which leads to muscle tension and this again exacerbates the development of pain on the MSDS. Conversely, satisfied restaurant workers could manage the job demand, control imbalance in a better way, and minimize the risk of WMSDs than their counterparts. However, studies conducted in Bangladesh [23], Turkey [22], and Taiwan [19] were not statistically significant.

Though this study was able to provide important information on the WMSDs among restaurant workers, it shares the following limitations. First, ergonomics hazard assessment and measurement of working environment tools were not used to assess WMSDs. This could result in over or underestimation of the findings. Secondly, there could be recall bias since data was collected within a year. Thirdly, it is difficult to establish a temporal relationship because of the cross-sectional nature of the data.

Work-related factors contributing to the conditions pretending the government and other stakeholders could benefit from it and it will also help as a baseline for policymakers, researchers, and implementations of occupational health and safety service.

## 5. Conclusion

In this study, the prevalence of WMSDs among restaurant workers was high. The upper back, lower back, elbow, and wrist were the most affected body parts. Age above 30 years, educational status being primary and secondary, and being dissatisfied by their job were positively associated with a high prevalence of the WMSDs.

## Figures and Tables

**Table 1:** Sociodemographic characteristics of restaurant workers in Gondar city, Ethiopia, April 2020 (*N* = 595).

Variable	Frequency (*n*)	Percent (%)
*Sex*		
Female	419	70.42
Male	176	29.58

*Age*		
≤30	524	88.07
>30	71	11.93

*Marital status*		
Single	428	71.93
Married	143	24.03
Divorced/widowed	24	4.04

*Religion*		
Orthodox	585	98.32
Muslim	3	0.5
Protestant	7	1.18

*Educational status*		
Illiterate	15	2.52
Able to read/write	5	0.84
Primary (1–8 grades)	167	28.07
Secondary (9–12 grades)	218	36.64
Higher education	190	31.93

*Monthly salary*		
≤1100 ETB	310	52.10
1101–1700 ETB	143	24.03
>1700 ETB	142	13.87

*Specific work experience*		
1–2 years	330	55.46
>2 years	265	44.54

**Table 2:** Personal characteristics of restaurant workers in Gondar city, Ethiopia, April 2020 (*N* = 595).

Variable	Frequency (*n*)	Percent (%)
*Smoking cigarette*		
Yes	5	590
No	0.84	99.16

*Alcohol drinking*		
Yes	104	16.97
No	494	83.03

*Physical exercise*		
Yes	105	17.65
No	490	82.35

*BMI*		
Under	25	4.2
Normal	482	81.01
Over	88	14.79

*Dominant hand*		
Right hand	576	96.81
Left hand	19	3.19

*Job training*		
Yes	147	24.71
No	448	75.29

*Ergonomic training*		
Yes	142	23.87
No	453	76.13

**Table 3:** Workplace characteristics of restaurant workers in Gondar city, Ethiopia, April 2020 (*N* = 595).

Variables	Frequency (*n*)	Percent (%)
*Bending or twisting in an awkward way*		
Yes	427	71.76
No	168	28.24

*Hours of bending/twisting in an awkward way* (*n* = 427)		
1–3 hours	276	64.64
4–6 hours	151	35.36

*Work in a static posture*		
Yes	392	65.88
No	203	34.12

*Highly repetitive work*		
Yes	427	71.76
No	168	28.24

*Working hours per day*		
≤8 hours	283	47.56
>8 hours	312	52.44

*Working days per week*		
4–6 days	171	28.74
7 days	424	71.26

*Extra break excluding lunchtime*		
Yes	99	16.64
No	496	83.36

*Times taking break excluding lunchtime* (*n* = 99)		
1–2 times	73	73.74
>3 times	26	26.26

*Number of customers in the restaurants*		
≤100	336	56.47
101–250	220	36.97
251–800	39	6.55

**Table 4:** Working environment and psychosocial characteristics of restaurant workers in Gondar city, Ethiopia, April 2020 (*n* = 595).

Variables	Frequency (*n*)	Percent (%)
*Adjustable chair* (*table*)		
Yes	332	55.8
No	263	44.2

*Colleague relationship*		
Good	394	66.2
Fair	201	33.8

*Customers relationship*		
Good	432	72.61
Fair	163	27.39

*Boss relationship*		
Good	439	73.78
Fair	146	24.54
Poor	10	1.68

*Safety training*		
Yes	214	35.97
No	381	64.03

*Thermal comfort*		
No thermal	80	13.45
Has thermal	515	86.55

*Job stress*		
No stress	57	9.58
Has stress	538	90.42

*Job satisfaction*		
Not satisfied	490	82.35
Has satisfied	105	17.65

**Table 5:** Multiple body parts WMSDs in body segments among restaurant workers in Gondar city, Ethiopia, April 2020 (*N* = 595).

Body parts	Numbers (*n*)	Percent (%)
*Shoulder*		
Both	147	24.7
Right	84	14.1
Left	35	5.88
No	329	55.3

*Elbow*		
Both	233	39.2
Right	35	5.88
Left	52	8.74
No	275	46.2

*Wrist*		
Both	241	40.5
Right	40	6.72
Left	26	4.37
No	288	48.4

*Hips/tights*		
Both	115	19.33
Right	38	6.38
Left	47	7.9
No	395	66.4

*Knee*		
Both	173	29.1
Right	31	5.21
Left	38	6.4
No	353	59.3

*Ankle*		
Both	201	33.78
Right	19	3.2
Left	26	4.36
No	349	58.66

**Table 6:** Self-adopted management strategies for musculoskeletal complaints of restaurant workers in Gondar city, April 2020 (*N* = 595).

Variables	Frequency	Percent
*Did not do anything*		
Yes	154	25.88
No	441	74.12

*Taking sufficient rest*		
Yes	176	29.58
No	419	70.42

*Reduced working hours*		
Yes	95	15.97
No	500	84.03

*Visited a physician*		
Yes	104	17.48
No	491	82.52

*Stop attending work if it causes or worsens discomfort*		
Yes	93	15.63
No	502	84.37

*Modifying the positions to be comfortable*		
Yes	126	21.18
No	469	78.82

*Take homemade management*		
Yes	164	27.56
No	431	72.44

**Table 7:** Bivariate and multivariable binary logistic regression analysis of factors associated with WMSDs among restaurant workers, Gondar city, 2020 (*N* = 595).

Variable		WMSDs		COR (95%CI)	AOR (95%CI)
		Yes	No		
*Age*	≤30	418	106	1	1
	>30	67	4	4.24 (1.51–11.91)^*∗*^	4.7 (1.62–13.84)^*∗*^

*Educational status*	Illiterate and read/write	17	3	1.81 (0.5–6.6)	1.79 (0.47–6.78)
	Primary (1–8 grades)	145	22	2.1 (1.21–3.77)^*∗*^	2.14 (1.17–3.90)^*∗*^
	Secondary (9–12 grades)	180	38	1.55 (0.96–2.51)	1.71 (1.02–2.86)^*∗*^
	Higher education	143	47	1	1

*Monthly salary*	<1100	256	54	1.21 (0.74–2.01)	1.27 (0.723–2.23)
	1101–1700	116	27	1.10 (0.61–1.1.97)	1.22 (0.65–2.26)
	>1701	113	29	1	1

*Specific work experience*	1–2 years	267	63	1	1
	>2 years	218	47	1.1 (0.73–1.67)	0.96 (0.61–1.52)

*BMI*	Under	20	5	0.93 (0.34–2.54)	1.31 (0.46–3.72)
	Normal	391	91	1	1
	Overweight	74	14	1.23 (0.59–2.22)	1.14 (0.60–2.19)

*Bending/twisting*	Yes	354	73	1.8 (1.19–2.85)^*∗*^	0.91 (0.54–1.52)
	No	131	37	1	1

*Work repetition*	Yes	357	70	1.59 (1.02–2.43)^*∗*^	1.25 (0.76–2.05)
	No	128	40	1	1

*Adjustable chair* (*table*)	Yes	262	70	1	1
	No	223	40	1.43 (0.93–2.18)	1.18 (0.74–1.89)

Job satisfaction	No satisfaction	407	83	1.69 (1.03–2.80)^*∗*^	1.90 (1.13–3.19)^*∗*^
	Has satisfaction	78	27	1	1

## Data Availability

The data will be made available from the primary author or corresponding author upon a reasonable request. The data contain indirect identifying characteristics (e.g., age and sex). The data are available on requests to the primary author (Habtamu Tegenu at habtie184@gmail.com) or the corresponding author (Jember Azanaw at jemberazanaw21@gmail.com) at the University of Gondar.

## Abbreviations

AOR: Adjusted odds ratio
BMI: Body mass index
CI: Confidence interval
COR: Crude odds ratio
ETB: Ethiopian birr
HSE: Health and safety executive
ILO: International Labor Organization
LBP: Lower back pain
MSDs: Musculoskeletal disorders
OHS: Occupational health and safety
OR: Odds ratio
PPE: Personal protective equipment
SD: Standard deviation
UBP: Upper back pain
US: United State
WHO: World Health Organization
WMSDs: Work-related musculoskeletal disorders.
